# 3,5-Dimeth­oxy-*N*,*N*-bis­(2-pyridylmeth­yl)aniline

**DOI:** 10.1107/S1600536809048685

**Published:** 2009-11-21

**Authors:** Hongjuan Li, Xianping Dai, Jufeng Sun

**Affiliations:** a School of Pharmacy, Binzhou Medical College, Yantai 264003, People’s Republic of China

## Abstract

In the title mol­ecule, C_20_H_21_N_3_O_2_, the benzene ring forms dihedral angles of 80.8 (1) and 83.5 (1)° with the two terminal pyridine rings. In the crystal structure, weak inter­molecular C—H⋯O hydrogen bonds link the mol­ecules into chains propagating in [001].

## Related literature

For general background to organic ligand-based crystal mater­ials, see: Desiraju (2007 ▶); Moulton & Zaworotko (2001 ▶). For related structures, see: Frisch & Cahil (2008 ▶); Shattock *et al.* (2008 ▶); Shirman *et al.* (2008 ▶).
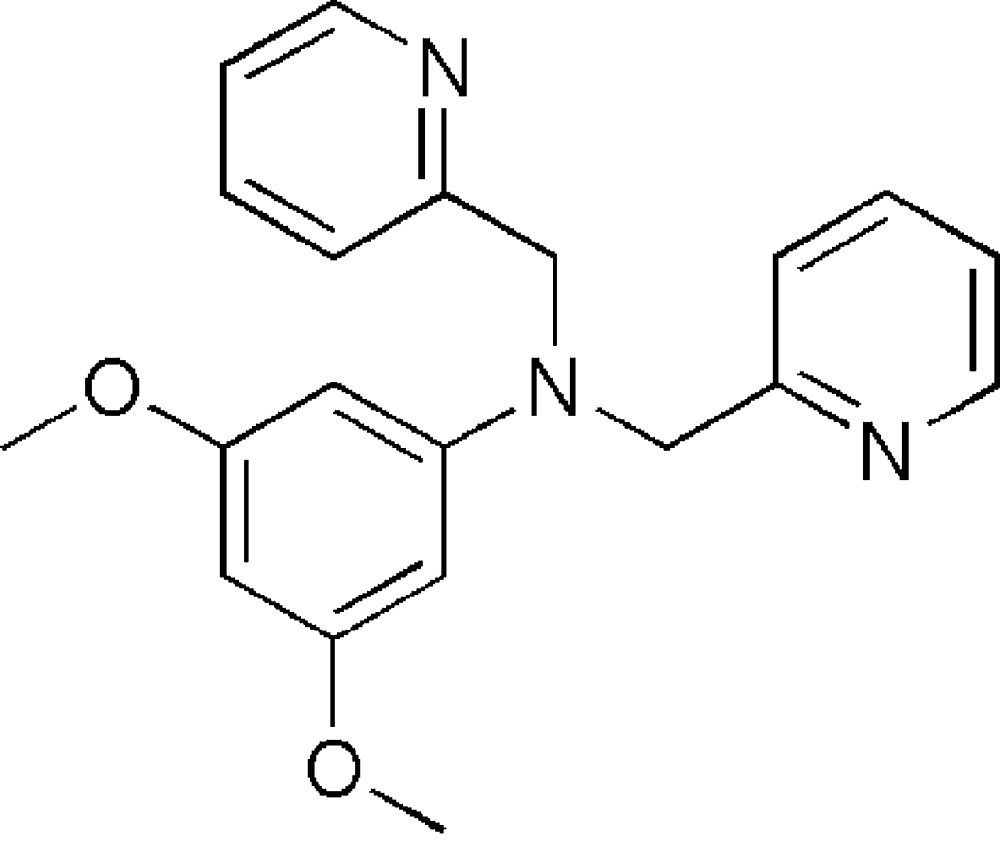


## Experimental

### 

#### Crystal data

C_20_H_21_N_3_O_2_
*M* *_r_* = 335.40
Monoclinic, 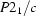

*a* = 15.630 (3) Å
*b* = 5.9562 (12) Å
*c* = 20.088 (4) Å
β = 111.55 (3)°
*V* = 1739.3 (6) Å^3^
*Z* = 4
Mo *K*α radiation
μ = 0.08 mm^−1^
*T* = 113 K
0.27 × 0.25 × 0.20 mm

#### Data collection

Rigaku Saturn CCD area-detector diffractometer
Absorption correction: multi-scan (*CrystalClear*; Rigaku/MSC, 2005 ▶) *T*_min_ = 0.978, *T*_max_ = 0.983
14749 measured reflections
4106 independent reflections
3258 reflections with *I* > 2σ(*I*)
*R*_int_ = 0.038

#### Refinement

*R*[*F* ^2^ > 2σ(*F* ^2^)] = 0.041
*wR*(*F* ^2^) = 0.113
*S* = 1.10
4106 reflections
228 parameters
H-atom parameters constrained
Δρ_max_ = 0.22 e Å^−3^
Δρ_min_ = −0.21 e Å^−3^

### 

Data collection: *CrystalClear* (Rigaku/MSC, 2005 ▶); cell refinement: *CrystalClear*; data reduction: *CrystalClear*; program(s) used to solve structure: *SHELXS97* (Sheldrick, 2008 ▶); program(s) used to refine structure: *SHELXL97* (Sheldrick, 2008 ▶); molecular graphics: *ORTEPIII* (Burnett & Johnson, 1996 ▶); software used to prepare material for publication: *SHELXL97* and *PLATON* (Spek, 2009 ▶).

## Supplementary Material

Crystal structure: contains datablocks global, I. DOI: 10.1107/S1600536809048685/cv2657sup1.cif

Structure factors: contains datablocks I. DOI: 10.1107/S1600536809048685/cv2657Isup2.hkl

Additional supplementary materials:  crystallographic information; 3D view; checkCIF report

## Figures and Tables

**Table 1:** Hydrogen-bond geometry (Å, °)

*D*—H⋯*A*	*D*—H	H⋯*A*	*D*⋯*A*	*D*—H⋯*A*
C14—H14⋯O2^i^	0.95	2.49	3.3050 (15)	144

Symmetry code: (i) 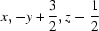.

**Fig. 1.**
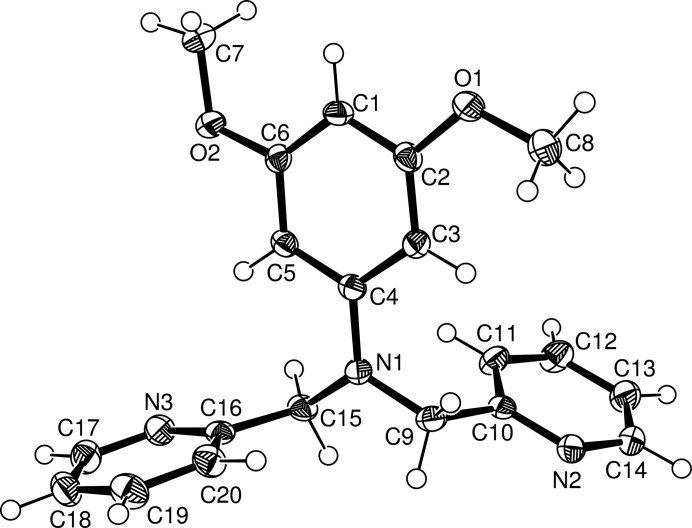
The molecular structure of (I), showing 50% probability displacement ellipsoids and the atomic numbering.

## supplementary crystallographic information

### Comment

In recent years, considerable research has been put into the design and elaboration of new organic ligand-based crystal materials because of their importance in supramolecular chemistry, materials science and solid-state chemistry (Desiraju, 2007; Moulton & Zaworotko, 2001). It is well known that the construction of such materials strongly depends on the nature of organic bridging units. In this regard, considerable attention has been devoted to the design of new functional *N*-heterocyclic organic bridging units. Among of them, pyridines are useful building blocks, which are frequently employed in the construction of some interesting metal-organic frameworks and organic crystals (Frisch & Cahil, 2008; Shattock *et al.*, 2008; Shirman *et al.*, 2008). Herein, we report a new pyridine compound which could be applied for the preparation of metal-organic and organic crystals.

In the title molecule, (I) (Fig. 1), two pyridine rings form dihedral angles of 80.8 (1) and 83.5 (1)°, respectively, with the central benzene ring. The intermolecular C—H···O interaction (Table 1) links adjacent molecules into chains along the direction [001].

### Experimental

3,5-Dimethoxyaniline (73.9 mg, 0.6 mmol) and 5 N NaOH (0.8 ml) were added to the solution of 2-bromomethylprydine (0.525 g, 3.05 mmol) in 1 ml of water, the obtained mixture was stirred vigorously for 24 h at room temperature. Then the mixture was extracted with 15 ml of CH_2_Cl_2_ for three times and the combined organic layers were dried over anhydrous Na_2_SO_4_. The crude material was purified by column chromatography on silica gel eluting with petroleum ether/EtOAc (3/1, V/V) to afford the desired product as a yellow solid (0.12 g, 58%). ^1^H NMR (400 MHz, CDCl_3_): δ = 3.64 (s, 6H), 4.82 (s, 4H), 5.87 (s, 3H), 7.14 (t, *J* = 6.4 Hz, 2H), 7.29 (s, 2H), 7.64 (t, *J* = 7.6 Hz, 2H), 8.58 (d, *J* = 4.4 Hz, 2H).

### Refinement

All H atoms were positioned geometrically (C—H 0.95 - 0.99 Å), and refined in the riding model approximation, with U_iso_(H) = 1.2-1.5 U_eq_(C) .

### Crystal data

**Table:**

C_20_H_21_N_3_O_2_	*F*(000) = 712
*M**_r_* = 335.40	*D*_x_ = 1.281 Mg m^−^^3^
Monoclinic, *P*2_1_/*c*	Mo *K*α radiation, λ = 0.71073 Å
Hall symbol: -P 2ybc	Cell parameters from 4823 reflections
*a* = 15.630 (3) Å	θ = 2.2–27.9°
*b* = 5.9562 (12) Å	µ = 0.08 mm^−^^1^
*c* = 20.088 (4) Å	*T* = 113 K
β = 111.55 (3)°	Block, colourless
*V* = 1739.3 (6) Å^3^	0.27 × 0.25 × 0.20 mm
*Z* = 4	

### Data collection

**Table:**

Rigaku Saturn CCD area-detector diffractometer	4106 independent reflections
Radiation source: rotating anode	3258 reflections with *I* > 2σ(*I*)
confocal	*R*_int_ = 0.038
Detector resolution: 7.31 pixels mm^-1^	θ_max_ = 27.9°, θ_min_ = 2.1°
ω and φ scans	*h* = −20→13
Absorption correction: multi-scan (*CrystalClear*; Rigaku/MSC, 2005)	*k* = −7→7
*T*_min_ = 0.978, *T*_max_ = 0.983	*l* = −26→26
14749 measured reflections	

### Refinement

**Table:**

Refinement on *F*^2^	Primary atom site location: structure-invariant direct methods
Least-squares matrix: full	Secondary atom site location: difference Fourier map
*R*[*F*^2^ > 2σ(*F*^2^)] = 0.041	Hydrogen site location: inferred from neighbouring sites
*wR*(*F*^2^) = 0.113	H-atom parameters constrained
*S* = 1.10	*w* = 1/[σ^2^(*F*_o_^2^) + (0.0655*P*)^2^] where *P* = (*F*_o_^2^ + 2*F*_c_^2^)/3
4106 reflections	(Δ/σ)_max_ = 0.001
228 parameters	Δρ_max_ = 0.22 e Å^−^^3^
0 restraints	Δρ_min_ = −0.21 e Å^−^^3^

### Special details

**Table:**

Geometry. All e.s.d.'s (except the e.s.d. in the dihedral angle between two l.s. planes) are estimated using the full covariance matrix. The cell e.s.d.'s are taken into account individually in the estimation of e.s.d.'s in distances, angles and torsion angles; correlations between e.s.d.'s in cell parameters are only used when they are defined by crystal symmetry. An approximate (isotropic) treatment of cell e.s.d.'s is used for estimating e.s.d.'s involving l.s. planes.
Refinement. Refinement of *F*^2^ against ALL reflections. The weighted *R*-factor *wR* and goodness of fit *S* are based on *F*^2^, conventional *R*-factors *R* are based on *F*, with *F* set to zero for negative *F*^2^. The threshold expression of *F*^2^ > σ(*F*^2^) is used only for calculating *R*-factors(gt) *etc*. and is not relevant to the choice of reflections for refinement. *R*-factors based on *F*^2^ are statistically about twice as large as those based on *F*, and *R*- factors based on ALL data will be even larger.

### Fractional atomic coordinates and isotropic or equivalent isotropic displacement parameters (Å^2^)

**Table:**

	*x*	*y*	*z*	*U*_iso_*/*U*_eq_	
O1	0.46394 (5)	0.69311 (15)	0.32987 (5)	0.0298 (2)	
O2	0.67567 (5)	0.25014 (13)	0.51460 (4)	0.0262 (2)	
N1	0.79174 (6)	0.85369 (16)	0.41961 (5)	0.0225 (2)	
N2	0.76094 (6)	1.11713 (16)	0.24609 (5)	0.0232 (2)	
N3	0.98074 (6)	0.69084 (17)	0.58297 (5)	0.0270 (2)	
C1	0.56528 (7)	0.47409 (19)	0.42047 (6)	0.0225 (2)	
H1	0.5142	0.3882	0.4208	0.027*	
C2	0.55351 (7)	0.65475 (19)	0.37374 (6)	0.0223 (2)	
C3	0.62677 (7)	0.78159 (19)	0.37172 (6)	0.0215 (2)	
H3	0.6168	0.9019	0.3387	0.026*	
C4	0.71674 (7)	0.72922 (18)	0.41968 (6)	0.0195 (2)	
C5	0.72901 (7)	0.55021 (18)	0.46686 (6)	0.0203 (2)	
H5	0.7889	0.5140	0.4995	0.024*	
C6	0.65369 (7)	0.42403 (18)	0.46634 (6)	0.0204 (2)	
C7	0.60170 (8)	0.1182 (2)	0.51853 (7)	0.0298 (3)	
H7A	0.5602	0.2138	0.5326	0.045*	
H7B	0.6261	−0.0012	0.5540	0.045*	
H7C	0.5679	0.0513	0.4716	0.045*	
C8	0.44478 (8)	0.8805 (2)	0.28219 (7)	0.0334 (3)	
H8A	0.4656	1.0189	0.3098	0.050*	
H8B	0.3784	0.8893	0.2550	0.050*	
H8C	0.4772	0.8618	0.2490	0.050*	
C9	0.78119 (8)	1.03228 (18)	0.36833 (6)	0.0226 (2)	
H9A	0.8346	1.1346	0.3873	0.027*	
H9B	0.7254	1.1196	0.3637	0.027*	
C10	0.77348 (7)	0.95312 (18)	0.29448 (6)	0.0196 (2)	
C11	0.78065 (8)	0.72861 (19)	0.27911 (6)	0.0260 (3)	
H11	0.7887	0.6166	0.3145	0.031*	
C12	0.77589 (9)	0.6707 (2)	0.21078 (7)	0.0317 (3)	
H12	0.7814	0.5183	0.1990	0.038*	
C13	0.76299 (9)	0.8373 (2)	0.16036 (6)	0.0308 (3)	
H13	0.7597	0.8028	0.1133	0.037*	
C14	0.75496 (8)	1.0555 (2)	0.18028 (6)	0.0271 (3)	
H14	0.7445	1.1696	0.1451	0.033*	
C15	0.88451 (7)	0.7731 (2)	0.45984 (6)	0.0234 (3)	
H15A	0.9269	0.8364	0.4383	0.028*	
H15B	0.8854	0.6077	0.4553	0.028*	
C16	0.91979 (7)	0.83373 (19)	0.53877 (6)	0.0210 (2)	
C17	1.01752 (8)	0.7486 (2)	0.65253 (7)	0.0306 (3)	
H17	1.0602	0.6481	0.6846	0.037*	
C18	0.99727 (8)	0.9438 (2)	0.68009 (7)	0.0317 (3)	
H18	1.0265	0.9791	0.7294	0.038*	
C19	0.93321 (8)	1.0875 (2)	0.63410 (7)	0.0327 (3)	
H19	0.9168	1.2229	0.6514	0.039*	
C20	0.89329 (8)	1.0310 (2)	0.56236 (6)	0.0272 (3)	
H20	0.8484	1.1260	0.5298	0.033*	

### Atomic displacement parameters (Å^2^)

**Table:**

	*U*^11^	*U*^22^	*U*^33^	*U*^12^	*U*^13^	*U*^23^
O1	0.0161 (4)	0.0381 (5)	0.0312 (5)	−0.0010 (3)	0.0040 (3)	0.0086 (4)
O2	0.0244 (4)	0.0261 (5)	0.0271 (4)	−0.0037 (3)	0.0083 (3)	0.0054 (3)
N1	0.0175 (5)	0.0297 (5)	0.0204 (5)	−0.0037 (4)	0.0069 (4)	0.0031 (4)
N2	0.0220 (5)	0.0220 (5)	0.0235 (5)	−0.0002 (4)	0.0058 (4)	0.0019 (4)
N3	0.0217 (5)	0.0299 (6)	0.0281 (5)	0.0012 (4)	0.0075 (4)	0.0028 (4)
C1	0.0184 (5)	0.0268 (6)	0.0234 (6)	−0.0056 (4)	0.0090 (4)	−0.0015 (5)
C2	0.0177 (5)	0.0289 (6)	0.0200 (5)	0.0005 (4)	0.0066 (4)	−0.0014 (5)
C3	0.0208 (5)	0.0243 (6)	0.0199 (5)	−0.0005 (4)	0.0082 (4)	0.0012 (4)
C4	0.0188 (5)	0.0233 (6)	0.0182 (5)	−0.0030 (4)	0.0089 (4)	−0.0046 (4)
C5	0.0166 (5)	0.0248 (6)	0.0190 (5)	−0.0006 (4)	0.0058 (4)	−0.0023 (4)
C6	0.0242 (6)	0.0210 (6)	0.0173 (5)	−0.0013 (4)	0.0093 (4)	−0.0018 (4)
C7	0.0315 (7)	0.0280 (6)	0.0317 (7)	−0.0062 (5)	0.0137 (5)	0.0036 (5)
C8	0.0237 (6)	0.0366 (7)	0.0342 (7)	0.0031 (5)	0.0040 (5)	0.0092 (6)
C9	0.0227 (5)	0.0220 (6)	0.0248 (6)	−0.0050 (4)	0.0109 (5)	−0.0017 (5)
C10	0.0147 (5)	0.0217 (6)	0.0220 (5)	−0.0023 (4)	0.0062 (4)	0.0003 (4)
C11	0.0338 (6)	0.0201 (6)	0.0268 (6)	−0.0021 (5)	0.0142 (5)	0.0030 (5)
C12	0.0460 (7)	0.0209 (6)	0.0321 (7)	−0.0032 (5)	0.0190 (6)	−0.0039 (5)
C13	0.0400 (7)	0.0305 (7)	0.0235 (6)	−0.0057 (5)	0.0135 (5)	−0.0032 (5)
C14	0.0300 (6)	0.0262 (6)	0.0218 (6)	−0.0036 (5)	0.0056 (5)	0.0039 (5)
C15	0.0162 (5)	0.0305 (6)	0.0247 (6)	−0.0033 (4)	0.0091 (4)	−0.0020 (5)
C16	0.0143 (5)	0.0239 (6)	0.0252 (6)	−0.0033 (4)	0.0076 (4)	0.0006 (5)
C17	0.0214 (6)	0.0402 (7)	0.0278 (6)	0.0024 (5)	0.0062 (5)	0.0070 (6)
C18	0.0232 (6)	0.0474 (8)	0.0228 (6)	−0.0051 (5)	0.0065 (5)	−0.0037 (6)
C19	0.0307 (7)	0.0334 (7)	0.0347 (7)	−0.0018 (5)	0.0129 (6)	−0.0090 (6)
C20	0.0217 (6)	0.0277 (6)	0.0297 (6)	0.0024 (5)	0.0063 (5)	0.0003 (5)

### Geometric parameters (Å, °)

**Table:**

O1—C2	1.3719 (14)	C8—H8B	0.9800
O1—C8	1.4290 (15)	C8—H8C	0.9800
O2—C6	1.3733 (13)	C9—C10	1.5185 (15)
O2—C7	1.4237 (14)	C9—H9A	0.9900
N1—C4	1.3872 (14)	C9—H9B	0.9900
N1—C9	1.4475 (14)	C10—C11	1.3862 (16)
N1—C15	1.4577 (14)	C11—C12	1.3905 (17)
N2—C10	1.3408 (14)	C11—H11	0.9500
N2—C14	1.3417 (15)	C12—C13	1.3788 (17)
N3—C16	1.3411 (15)	C12—H12	0.9500
N3—C17	1.3458 (16)	C13—C14	1.3794 (18)
C1—C6	1.3827 (16)	C13—H13	0.9500
C1—C2	1.3949 (16)	C14—H14	0.9500
C1—H1	0.9500	C15—C16	1.5185 (16)
C2—C3	1.3847 (15)	C15—H15A	0.9900
C3—C4	1.4169 (16)	C15—H15B	0.9900
C3—H3	0.9500	C16—C20	1.3859 (16)
C4—C5	1.3923 (15)	C17—C18	1.3738 (18)
C5—C6	1.3935 (15)	C17—H17	0.9500
C5—H5	0.9500	C18—C19	1.3816 (18)
C7—H7A	0.9800	C18—H18	0.9500
C7—H7B	0.9800	C19—C20	1.3852 (17)
C7—H7C	0.9800	C19—H19	0.9500
C8—H8A	0.9800	C20—H20	0.9500
			
C2—O1—C8	118.28 (9)	N1—C9—H9B	108.6
C6—O2—C7	117.23 (9)	C10—C9—H9B	108.6
C4—N1—C9	121.20 (9)	H9A—C9—H9B	107.6
C4—N1—C15	119.49 (9)	N2—C10—C11	122.92 (10)
C9—N1—C15	117.81 (9)	N2—C10—C9	114.84 (9)
C10—N2—C14	116.99 (10)	C11—C10—C9	122.23 (10)
C16—N3—C17	116.98 (11)	C10—C11—C12	118.68 (11)
C6—C1—C2	117.86 (10)	C10—C11—H11	120.7
C6—C1—H1	121.1	C12—C11—H11	120.7
C2—C1—H1	121.1	C13—C12—C11	119.13 (12)
O1—C2—C3	123.36 (10)	C13—C12—H12	120.4
O1—C2—C1	114.32 (10)	C11—C12—H12	120.4
C3—C2—C1	122.31 (10)	C12—C13—C14	118.00 (11)
C2—C3—C4	119.01 (10)	C12—C13—H13	121.0
C2—C3—H3	120.5	C14—C13—H13	121.0
C4—C3—H3	120.5	N2—C14—C13	124.25 (11)
N1—C4—C5	120.32 (10)	N2—C14—H14	117.9
N1—C4—C3	120.64 (10)	C13—C14—H14	117.9
C5—C4—C3	119.04 (10)	N1—C15—C16	113.97 (9)
C4—C5—C6	120.21 (10)	N1—C15—H15A	108.8
C4—C5—H5	119.9	C16—C15—H15A	108.8
C6—C5—H5	119.9	N1—C15—H15B	108.8
O2—C6—C1	124.25 (10)	C16—C15—H15B	108.8
O2—C6—C5	114.19 (9)	H15A—C15—H15B	107.7
C1—C6—C5	121.56 (10)	N3—C16—C20	122.72 (11)
O2—C7—H7A	109.5	N3—C16—C15	115.94 (10)
O2—C7—H7B	109.5	C20—C16—C15	121.27 (10)
H7A—C7—H7B	109.5	N3—C17—C18	124.13 (11)
O2—C7—H7C	109.5	N3—C17—H17	117.9
H7A—C7—H7C	109.5	C18—C17—H17	117.9
H7B—C7—H7C	109.5	C17—C18—C19	118.20 (12)
O1—C8—H8A	109.5	C17—C18—H18	120.9
O1—C8—H8B	109.5	C19—C18—H18	120.9
H8A—C8—H8B	109.5	C18—C19—C20	118.94 (12)
O1—C8—H8C	109.5	C18—C19—H19	120.5
H8A—C8—H8C	109.5	C20—C19—H19	120.5
H8B—C8—H8C	109.5	C19—C20—C16	119.00 (11)
N1—C9—C10	114.49 (9)	C19—C20—H20	120.5
N1—C9—H9A	108.6	C16—C20—H20	120.5
C10—C9—H9A	108.6		
			
C8—O1—C2—C3	−3.27 (16)	C14—N2—C10—C11	0.28 (16)
C8—O1—C2—C1	177.50 (10)	C14—N2—C10—C9	179.13 (9)
C6—C1—C2—O1	179.80 (10)	N1—C9—C10—N2	178.10 (9)
C6—C1—C2—C3	0.56 (17)	N1—C9—C10—C11	−3.04 (15)
O1—C2—C3—C4	179.59 (10)	N2—C10—C11—C12	0.90 (17)
C1—C2—C3—C4	−1.24 (17)	C9—C10—C11—C12	−177.88 (10)
C9—N1—C4—C5	176.48 (9)	C10—C11—C12—C13	−0.87 (18)
C15—N1—C4—C5	10.78 (15)	C11—C12—C13—C14	−0.28 (18)
C9—N1—C4—C3	−3.31 (16)	C10—N2—C14—C13	−1.55 (17)
C15—N1—C4—C3	−169.01 (10)	C12—C13—C14—N2	1.56 (19)
C2—C3—C4—N1	−179.48 (10)	C4—N1—C15—C16	−83.43 (13)
C2—C3—C4—C5	0.72 (16)	C9—N1—C15—C16	110.39 (11)
N1—C4—C5—C6	−179.36 (10)	C17—N3—C16—C20	−1.19 (16)
C3—C4—C5—C6	0.44 (16)	C17—N3—C16—C15	175.75 (9)
C7—O2—C6—C1	−1.89 (16)	N1—C15—C16—N3	150.93 (10)
C7—O2—C6—C5	178.13 (10)	N1—C15—C16—C20	−32.08 (14)
C2—C1—C6—O2	−179.33 (10)	C16—N3—C17—C18	−0.83 (18)
C2—C1—C6—C5	0.65 (16)	N3—C17—C18—C19	1.91 (19)
C4—C5—C6—O2	178.84 (9)	C17—C18—C19—C20	−0.95 (18)
C4—C5—C6—C1	−1.15 (17)	C18—C19—C20—C16	−0.90 (18)
C4—N1—C9—C10	−79.37 (13)	N3—C16—C20—C19	2.06 (17)
C15—N1—C9—C10	86.56 (12)	C15—C16—C20—C19	−174.73 (10)

### Hydrogen-bond geometry (Å, °)

**Table:**

*D*—H···*A*	*D*—H	H···*A*	*D*···*A*	*D*—H···*A*
C14—H14···O2^i^	0.95	2.49	3.3050 (15)	144

Symmetry codes: (i) *x*, −*y*+3/2, *z*−1/2.
